# 1,25-dihydroxyvitamin-D3 but not the clinically applied marker 25-hydroxyvitamin-D3 predicts survival after stem cell transplantation

**DOI:** 10.1038/s41409-020-01031-w

**Published:** 2020-08-27

**Authors:** Katrin Peter, Peter J. Siska, Tobias Roider, Carina Matos, Heiko Bruns, Kathrin Renner, Katrin Singer, Daniela Weber, Martina Güllstorf, Nicolaus Kröger, Daniel Wolff, Wolfgang Herr, Francis Ayuk, Ernst Holler, Klaus Stark, Iris M. Heid, Marina Kreutz

**Affiliations:** 1 Department of Internal Medicine III, Hematology and Medical Oncology, University Medical Center of Regensburg, Regensburg, Germany; 2 Department of Internal Medicine 5—Hematology/Oncology, University Hospital of Erlangen, Erlangen, Germany; 3 Department of Stem Cell Transplantation, University Medical Center Hamburg-Eppendorf, Hamburg, Germany; 4 Department for Genetic Epidemiology, University of Regensburg, Regensburg, Germany; 5 Present Address: Department of Medicine V, University of Heidelberg, Heidelberg, Germany

**Keywords:** Haematological cancer, Haematological cancer

## Abstract

The serum level of 25-hydroxyvitamin-D3 is accepted as marker for a person’s vitamin D status but its role for the outcome of allogeneic hematopoietic stem cell transplantation (HSCT) is controversially discussed. The impact of 1,25-dihydroxyvitamin-D3 on HSCT outcome, however, has never been studied. In a discovery cohort of 143 HSCT patients we repeatedly (day −16 to 100) measured 1,25-dihydroxyvitamin-D3 and in comparison the well-established marker for serum vitamin D status 25-hydroxyvitamin-D3. Only lower 1,25-dihydroxyvitamin-D3 levels around HSCT (day −2 to 7, peritransplant) were significantly associated with higher 1-year treatment-related mortality (TRM) risk (Mann–Whitney U test, *P* = 0.001). This was confirmed by Cox-model regression without and with adjustment for baseline risk factors and severe acute Graft-versus-Host disease (aGvHD; unadjusted *P* = 0.001, adjusted *P* = 0.005). The optimal threshold for 1,25-dihydroxyvitamin-D3 to identify patients at high risk was 139.5 pM. Also in three replication cohorts consisting of altogether 365 patients 1,25-dihydroxyvitamin-D3 levels below 139.5 pM had a 3.3-fold increased risk of TRM independent of severe aGvHD compared to patients above 139.5 pM (Cox-model unadjusted *P* < 0.0005, adjusted *P* = 0.001). Our data highlight peritransplant 1,25-dihydroxyvitamin-D3 levels but not the commonly monitored 25-hydroxyvitamin-D3 levels as potent predictor of 1-year TRM and suggest to monitor both vitamin D metabolites in HSCT patients.

## Introduction

Although treatment-related mortality (TRM) was substantially reduced among patients undergoing allogeneic hematopoietic stem cell transplantation (HSCT) over the past decade [1], the risk for TRM still remains high. Up to now, it is not possible to individually predict TRM at time of transplantation independent of known pretransplant variables as disease-associated risk factors, donor relationship, and comorbidity. An issue of debate is vitamin D3. In some studies, the impact of 25-hydroxyvitamin-D3 serum levels on adverse events of allogeneic HSCT was evaluated, but results are inconsistent [2–6]. In one prospective trial, safety and efficacy of vitamin D3 supplementation after HSCT was evaluated and no significant impact on overall survival was found [7].

25-hydroxyvitamin-D3 is a precursor of the active metabolite 1,25-dihydroxyvitamin-D3. The conversion of 25-hydroxyvitamin-D3 to 1,25-dihydroxyvitamin-D3 is mainly performed by the kidney, but also by immune cells [8, 9]. Little is known about the role of 1,25-dihydroxyvitamin-D3 levels with regard to HSCT and aGvHD. In rat bone marrow transplantation, an analog of 1,25-dihydroxyvitamin D3, has been shown to prevent aGvHD [10]. In humans, polymorphisms of the vitamin D receptor, mediating the biological activity of 1,25-dihydroxyvitamin-D3, were shown to correlate with survival and aGvHD in HLA-matched sibling allogeneic HSCT [11]. Recently, Carrillo-Cruz et al. reported a reduced incidence of cGvHD in response to vitamin D supplementation of patients with a certain VDR genotype [12].

We thus set out to investigate the role of 1,25-dihydroxyvitamin-D3 and for comparison also 25-hydroxyvitamin-D3 levels measured repeatedly shortly before and during the first 100 days after HSCT on 1-year TRM in a discovery cohort of 143 patients. We then tested our findings in overall 365 patients from three different cohorts in order to replicate our findings in independent data and to generalize it for different clinical settings.

## Materials and methods

### Patient recruitment and eligibility criteria for the discovery cohort

Our discovery cohort consisted of patients eligible for matched unrelated or matched related HSCT at the Regensburg University Medical Center with HSCT between May 2012 and February 2015. Excluded from HSCT were patients with poor performance status (Karnofsky grade < 80, corresponding to ECOG ≥ 2), progressive underlying disease, pregnancy or breast-feeding, moderate or severe cardiac insufficiency, moderate or severe renal insufficiency (serum creatinine levels > 2 mg/dl), high age (>70 years), uncontrolled acute or chronic infection or patients with liver failure (serum bilirubin > 2 mg/dl, levels of alanine aminotransferase (ALT), or aspartate aminotransferase (AST)) ≤ 2x upper normal level). From the cohort of 172 patients that underwent HSCT in the noted time period, 13 patients with repeated HSCT were excluded obtaining a cohort of 159 patients.

### Transplantation and concomitant therapy for patients in the discovery cohort

Patients were admitted to hospital 6–16 days before HSCT. Conditioning regimens included chemotherapy with or without irradiation and started between 6 and 8 days prior to HSCT. Bone marrow or peripheral-blood mononuclear cells were not T-cell-depleted or cryopreserved before HSCT. For aGvHD prophylaxis, patients received one of two immunosuppressive regimens: cyclosporine and methotrexate, or cyclosporine and mycophenolate. In case of cyclosporine-associated toxicity, the latter was replaced by tacrolimus. Prior to HSCT (days −3 to −1), additional immunosuppressive prophylaxis with rabbit antithymocyte globulin (ATG, Grafalon) was administered in patients receiving grafts from matched unrelated donors. Initial aGVHD treatment consisted of prednisolone 1–2 mg/kg/day with budesonide added in the presence of gastrointestinal symptoms. For gastrointestinal decontamination, all patients received prophylactic rifaximin (200 mg twice a day) from start of conditioning until engraftment (absolute neutrophil count >500/ µL). Upon signs of infection before and after HSCT, patients were treated with systemic broad spectrum antibiotics. For routine antifungal prophylaxis, patients received fluconazole or posaconazole, for pneumocystis-pneumonia prophylaxis, patients received cotrimoxazole. Upon signs of fungal infection, patients were treated with systemic mold active antifungal agents. Following HSCT, patients were monitored in the transplant unit and discharged after 28 days if clinically possible and subsequently regularly monitored in an outpatient setting.

### Vitamin D supplementation and serum measurements in the patients of the discovery cohort

All HSCT recipients in the discovery cohort received oral high-dose vitamin D3 supplementation (Vigantol oil, 20.000 IU/ml, Merck) consisting of a 50.000 IU-dose upon admission to hospital (d-16 to d-6) followed by daily administration of 10.000 IU. To monitor 25-hydroxyvitamin-D3 and 1,25-dihydroxyvitamin-D3 serum levels, blood was drawn repeatedly during inpatient stay and thereafter during routine outpatient visits. To avoid hypercalcemia, serum calcium levels were assessed twice a week. The described supplementation dose was maintained until patients reached 25-hydroxyvitamin-D3 serum levels of 150–200 nmol/L with subsequent dose adjustment to avoid 25-hydroxyvitamin-D3 levels >150–200 nmol/L. Vitamin D levels were analyzed immediately or from sera stored at −80 °C by the Department of Clinical Chemistry, University Medical Center of Regensburg. From May 2012 to October 2014, 25-hydroxyvitamin-D3 serum levels were determined by means of chemiluminescence immunoassay according to the manufacturer’s instructions (Immunodiagnostic systems, Frankfurt am Main, Germany). After validation for comparability, from November 2014 on, 25-hydroxyvitamin-D3 serum levels were analyzed by means of liquid chromatography high-resolution tandem mass spectrometry as described elsewhere [13]. 1,25-dihydroxyvitamin-D3 concentrations were measured using a radioimmunoassay according to the manufacturer’s instructions (Immunodiagnostic systems, Frankfurt am Main, Germany) by the Department of Clinical Chemistry, University Medical Center of Regensburg.

### Analyzed data for the discovery cohort

All of the 159 patients with full information on age, sex, donor type, survival, and available peritransplant 1,25-dihydroxyvitamin-D3 and 25-hydroxyvitamin-D3 levels were included into the analysis of the discovery cohort. Due to these criteria, further 16 patients with missing information about vitamin D serum levels at the day of HSCT were excluded obtaining a discovery cohort of 143 patients. Our primary endpoint was TRM within 1 year, defined as death due to any cause (aGvHD, infection, bleeding and organ failure) in the absence of relapse. Competing risk of death was defined as death due to relapse of the underlying malignancy. Overall mortality was defined as any death (TRM or relapse) within the first year. Mortality after the first year was not incorporated into our analysis. Our secondary endpoint was severe aGvHD, defined as aGvHD grade 3–4, within the first year according to clinical criteria [14].

For the analysis, serum 1,25-dihydroxyvitamin-D3 and 25-hydroxyvitamin-D3 values were categorized according to the time of blood sampling: at hospital admission (day −16 to −6, baseline), around HSCT (day −2 to 7, peritransplant), early follow-up around day 14 (day 11 to 17), day 21 (day 18 to 24), day 28 (day 25 to 31), and late follow-up (day 32 to 100). Missing baseline levels for 1,25-dihydroxyvitamin-D3 or 25-hydroxyvitamin-D3 were observed for 16 or 19, respectively, out of 143 patients; these were imputed by the median of existing 1,25-dihydroxyvitamin-D3 or 25-hydroxyvitamin-D3 baseline levels (82 pM or 43.5 nM, respectively). In the discovery cohort, median levels of 1,25-dihydroxyvitamin-D3 and 25-hydroxyvitamin-D3 at baseline were 172.0 pM and 54.0 nM, respectively. At the time of HSCT (day −2 to 7), at least one serum value for 1,25-dihydroxyvitamin-D3 and for 25-hydroxyvitamin-D3 was available for each patient due to our inclusion criteria. If multiple serum values within baseline, peritransplant or early follow-up intervals (until day 31) were available, the measurement closest to day −10, 0, 14, 21, or 28, respectively, was used. For the late follow-up (day 32 to 100), the median of the available 1,25-dihydroxyvitamin-D3 or 25-hydroxyvitamin-D3 serum levels, respectively, was used.

### Association analysis in discovery data

To avoid any model assumptions in our first analysis of the discovery data, the Mann–Whitney U test was applied to test 1,25-dihydroxyvitamin-D3 and 25-hydroxyvitamin-D3 levels at each time interval for differences between patients experiencing TRM compared to those who did not. We selected the metabolite and time intervals with statistically significant association with TRM (Bonferroni-corrected significance level at 0.05/(6 × 2) = 0.004).

For the selected metabolite and the time interval, we evaluated the association with time-to-death further and derived hazard ratio (HR) estimates using Cox proportional hazards models unadjusted and adjusted for risk factors (age, donor type, tumor stage prior to HSCT, intensity of the conditioning regime, severe aGvHD).

A Kaplan–Meier curve was generated to visualize 1-year-survival comparing patients with above versus below median serum vitamin D3 levels in the selected time intervals. The log rank test and an unadjusted Cox-model were used to test for difference in survival for patients with above versus below median serum levels. A receiver-operating-characteristic (ROC) curve and the optimal threshold for 1,25-dihydroxyvitamin-D3 serum levels for a best prediction of 1-year TRM were computed using the Youden Index [15]. All data analyses were performed using SPSS Statistics version 23 (IBM, Armonk, USA).

### Replication cohorts

Our replication stage consisted of three patient cohorts from various clinical settings to replicate our initial findings and to generalize for other clinical settings: (I) HSCT patients from Regensburg transplanted between March 2015 and May 2017 receiving the same high-dose vitamin D3 supplementation as the discovery cohort, (II) HSCT patients from Regensburg transplanted between March 2011 and February 2013 receiving vitamin D3 supplementation at lower dose (ranging from 1000 to 5000 IU/d, Vigantoletten, 1000 IU/tablet, Merck), (III) HSCT patients from the University Medical Center Hamburg-Eppendorf transplanted between February 2012 and August 2014 receiving no vitamin D3 supplementation. Eligibility and exclusion criteria for all three replication groups were the same as in the discovery cohort. We excluded 26 patients with unavailable serum aliquots from blood drawn at the relevant time interval, yielding *n* = 115, *n* = 107 and *n* = 143 patients in replication cohort I, II, and III, respectively.

In replication cohort I, concomitant therapy was the same as in the discovery cohort. For replication cohorts II and III, the concomitant therapy differed: in replication cohort II, the concomitant therapy was comparable to the discovery cohort with the exception of the administration of prophylactic Ciprofloxacin (500 mg twice a day) and Metronidazole (400 mg thrice a day) for gastrointestinal decontamination from start of conditioning until engraftment. Ciprofloxacin (500 mg twice a day) and Metronidazole (400 mg thrice a day) for gastrointestinal decontamination were also administered to 85 out of 143 patients in replication cohort III from start of conditioning until engraftment. In replication cohort III, ATG was administered to almost all patients independent of the source of transplanted cells (*n* = 117 rabbit ATG, Grafalon; *n* = 17 rabbit ATG, Merieux; *n* = 9 no ATG). Furthermore, initial aGvHD was treated systemically with Methyl-Prednisolone or Dexamethason and topically with topical steroids.

In all three replication cohorts, serum was stored at −80 °C and the serum obtained in the relevant time interval was transferred to Regensburg for central measurement. 25-hydroxyvitamin-D3 serum levels were analyzed as mentioned above. 1,25-dihydroxyvitamin-D3 levels were analyzed by the MVZ Laborzentrum Ettlingen, Germany, using the same method as described above.

### Association analysis in the replication stage

We utilized the optimal cutoff for vitamin D levels derived in the discovery data to define two groups of patients in the replication data: patients below and above the cutoff. We visualized survival in these two groups by Kaplan–Meier curves in each of the three cohorts and in the joint replication data.

We tested these dichotomized vitamin D levels for association with TRM in the joint replication data with the unadjusted Cox-model. We also applied a Cox-model unadjusted and adjusted for risk factors (age, donor type, tumor stage prior to HSCT, intensity of the conditioning regime, severe aGvHD). For this, we derived estimates for each covariate in the model separately by replication cohort and derived joint estimates across the three replication cohorts by a fixed-effect inverse-variance meta-analysis, for each covariate in the model. This joint estimate is an estimate of the underlying true effect, when all cohorts have the same effect, and it can be interpreted as the average of underlying effects, when the cohorts have varying effects [16].

### Study approval

The studies of the discovery cohort as well as the replication cohorts I and II were approved by the Ethics Committee of the University Medical Center of Regensburg (02/220). The study of the replication cohort III was approved by the Ethics Committee of the University Medical Center Hamburg-Eppendorf (PV4085). All subjects gave written informed consent according to internal standards and in accordance with the Declaration of Helsinki.

## Results

### Overview on design and collected patient samples

We adopted a two-stage design. In the discovery cohort of 143 patients (Regensburg study center, high-dose vitamin D3 supplementation), we tested 1,25-dihydroxyvitamin-D3 and 25-hydroxyvitamin-D3 levels measured at up to six time points between days −16 before HSCT up to day 100 after HSCT for their association with TRM within 1 year. Then, we selected the vitamin D metabolite and time interval showing statistical significant association with TRM for further analyses. Finally, we tested this metabolite from the selected time interval in independent replication data consisting of a total of 365 patients from three different clinic settings: (I) 115 patients from the same study center and the same setting as the discovery cohort (replication data I, Regensburg study center, high-dose vitamin D3 supplementation), (II) 107 patients from the same study center with an alternative type of vitamin D3 supplementation (replication data II, Regensburg study center, moderate vitamin D3 supplementation), and (III) 143 patients from another study center (replication data III, Hamburg study center, no vitamin D3 supplementation). All patients were enrolled at baseline shortly before HSCT (day −16 to −6) and followed for 365 days to record complications including severe aGvHD and survival status. Baseline patient characteristics of all 508 patients in this vitamin D3 study were similar across the four cohorts (Table 1). Median baseline vitamin D metabolite measurements across the four cohorts reflected the different approaches toward vitamin D3 supplementation (Table 2). Among the 508 patients, 89 patients experienced TRM and 70 died due to relapse or other causes (Table 2).

**Table 1:** Patient characteristics at baseline for the discovery and the three replication cohorts.

Characteristic	Discovery (*n* = 143)	Replication I (*n* = 115)	Replication II (*n* = 107)	Replication III (*n* = 143)
Study center	Regensburg	Regensburg	Regensburg	Hamburg
Vitamin D3 suppl.^a^	High-dose	High-dose	Moderate	None
Male sex	84 (58.7%)	75 (65.2%)	74 (69.2%)	94 (65.7%)
median age (range)[yr]	56 (26–70)	56 (19–69)	52 (19–71)	58 (18–75)
Diagnosis				
Aplastic anemia	2 (1.4%)	5 (4.3%)	2 (1.9%)	2 (1.4%)
Acute leukemia	76 (53.1%)	62 (53.9%)	55 (51.4%)	47 (32.9%)
Morbus Hodgkin	5 (3.5%)	0	1 (0.9%)	0
MDS	17 (11.9%)	16 (13.9%)	12 (11.2%)	25 (17.5%)
MPN	4 (2.8%)	12 (10.4%)	6 (5.6%)	27 (18.9%)
NHL	34 (23.8%)	18 (15.7%)	26 (24.3%)	20 (14.0%)
PMF	5 (3.5%)	2 (1.7%)	4 (3.7%)	22 (15.4%)
Late tumor stage^b^	75 (58.6%)	74 (64.3%)	65 (60.7%)	101 (70.6%)
Unrelated donor	102 (71.3%)	81 (70.4%)	83 (77.6%)	108 (75.5%)
ATG before HSCT yes	102 (71.3%)	81 (70.4%)	83 (77.6%)	134 (93.7%)
Standard conditioning^c^	13 (9.4%)	20 (17.4%)	17 (15.9%)	35 (24.5%)
Steroids ≥1 mg/kg^d^	65 (53.3%)	52 (45.2%)	55 (51.4%)	74 (51.7%)
Karnofsky score <90^e^	46 (33.3%)	53 (46.1%)	16 (15.0%)	94 (66%)

Shown are characteristics of the 508 patients in the vitamin D study. Stated are number of participants and percentages, if not indicated otherwise.

*suppl.* supplementation, *yr* years, *MDS* myelodysplastic syndrome, *MPN* myeloproliferative neoplasm, *NHL* non-Hodgkin lymphoma, *PMF* primary myelofibrosis, *HSCT* hematopoietic stem cell transplantation, *ATG* antithymocyte globulin.

^a^Type of vitamin D3 supplementation in the different cohorts: high-dose supplementation (50.000 IU p.o. at start, followed by 10.000 IU/d p.o.) was adjusted for patients with 25-hydroxyvitamin-D3 levels higher than 200 nmol/L; moderate supplementation: 1.000–5.000 IU/d p.o.

^b^At enrollment (before HSCT), no tumor stage grading available for 15 patients from discovery and for 1 patient from replication data I, classification according to EBMT risk score [50].

^c^Missing information on conditioning for discovery cohort from five patients.

^d^Missing information on steroid treatment for discovery cohort, replication cohort I, and replication cohort II from 21, 5, or 7 patients, respectively.

^e^Missing information on Karnofsky performance score for discovery cohort, replication cohort I, replication cohort II, and replication cohort III from 5, 8, 4, or 11 patients, respectively.

**Table 2:** Serum measurements and events during follow-up for the discovery and the three replication cohorts.

Measurement/event	Discovery (*n* = 143)	Replication I (*n* = 115)	Replication II (*n* = 107)	Replication III (*n* = 143)
Peritransplant 1,25(OH)2D3^a^				
*n*	143	115	107	143
Median [pM]	172.0	181.2	96.7	81.4
IQR [pM]	(97.0–238.0)	(132.6–259.2)	(62.5–161.9)	(53.0–128.0)
Peritransplant 25(OH)D3^b^				
*n*	143	48	69	141
Median [nM]	54.0	47.0	36.0	22.3
IQR [nM]	(42.0–67.0)	(38.3–64.0)	(29.0–49.7)	(14.8–38.2)
Mortality^c^				
TRM	23 (16.1%)	17 (14.8%)	24 (22.4%)	25 (17.5%)
Death due to relapse	17 (11.9%)	11 (9.6%)	21 (19.6%)	37 (25.9%)
Death, other causes	0	0	0	1
Lost-to-follow-up	0	0	0	0
None (alive after 1 yr)	109 (76.2%)	89 (77.4%)	72 (67.3%)	92 (64.3%)
#Severe aGvHD^d^	21 (14.7%)	9 (7.8%)	16 (15.0%)	18 (12.6%)
Median time to TRM^c^ [d after HSCT] (range)	163 (19–360)	165 (50–342)	118 (2–301)	113 (17–320)
IQR [d after HSCT]	89–342	57–242	43–192	56–198
Median time to max. severe aGvHD [d after HSCT] (range)	70 (14–354)	42 (27–220)	84 (17–275)	120 (5–331)
IQR [d after HSCT]	53–135	33–150	33–163	49–175

Shown are median vitamin D3 levels of the 508 patients in the vitamin D study and the number of deaths and other events occurring during follow-up. Stated are number of patients and percentages, if not indicated otherwise.

*1,25(OH)2D3* 1,25-dihydroxyvitamin-D3, *25(OH)D3* 25-hydroxyvitamin-D3, *IQR* interquartile range, *TRM* treatment-related mortality, *aGvHD* acute Graft-versus-Host disease, *HSCT* hematopoietic stem cell transplantation.

^a^1,25-dihydroxyvitamin-D3 level at time of HSCT (days −2 to 7).

^b^25-hydroxyvitamin-D3 level at time of HSCT (days −2 to 7).

^c^Death in first year after HSCT.

^d^Any occurrence of aGvHD 3–4 [14] during first year after HSCT; for replication cohort II from three patients evaluation of aGvHD grade not possible due to death early after HSCT or graft failure.

### Discovery cohort identified peritransplant 1,25-dihydroxyvitamin-D3 levels and late follow-up 25-hydroxyvitamin-D3 to predict first-year TRM

**Fig. 1:**
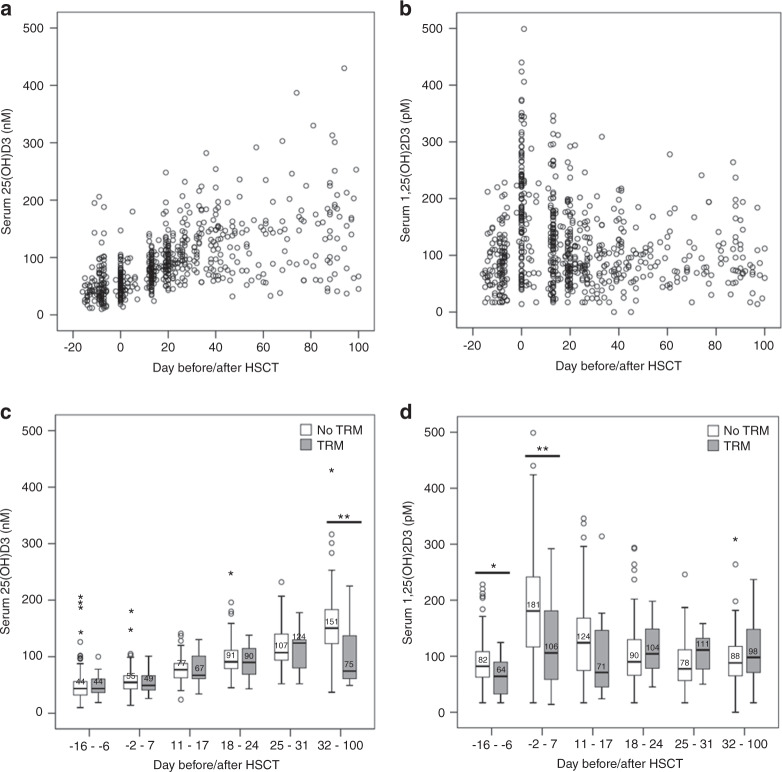
Time trend of 25-hydroxyvitamin-D3 and 1,25-dihydroxyvitamin-D3 serum levels and association with TRM in the discovery cohort. Shown are **a** 25-hydroxyvitamin-D3 (25(OH)D3) or **b** 1,25-dihydroxyvitamin-D3 (1,25(OH)2D3) serum levels in the discovery cohort that were measured repeatedly at hospital admission (baseline, day −16 to −6), peritransplant (day −2 to 7), during the weekly early follow-up (day 11 to 17, 18 to 24, 25 to 31) and the late follow-up (day 32 to 100 after HSCT) for 143 patients (details see Supplemental Table 1). Also shown is the distribution of **c** 25(OH)D3 **d** or 1,25(OH)2D3 levels per time interval separately for patients with or without TRM (23 patients with TRM, 120 patients without). Median serum levels are stated in bold for each boxplot; testing for difference of serum levels between the two groups was performed using Mann–Whitney U test, * or ** indicates a *P* value ≤0.05 (nominal significance) or ≤0.05/(6 × 2) = 0.004 (Bonferroni-corrected significance level), respectively (see Supplemental Table 1 for details).

In the discovery cohort of 143 patients, we first explored the time trend of serum levels from day −16 to day 100 after HSCT. 25-hydroxyvitamin-D3 revealed a slight, but steady increase, whereas 1,25-dihydroxyvitamin-D3 peaked around the time of HSCT (day −2 to 7, Fig. 1a, b). Further analysis was conducted with serum levels categorized into six different time intervals (Methods): (i) measurement at hospital admission (day −16 to −6, baseline), (ii) around HSCT (day −2 to 7, peritransplant), (iii, iv, v) early follow-up during the inpatient hospital stay with weekly samples (three intervals weekly apart: day 11 to 17; day 18 to 24; day 25 to 31), (vi) late follow-up with inpatient or outpatient care (days 32 to 100). Interval-specific median serum levels and number of deaths or severe aGvHD are shown in Supplemental Table S1. To identify association of serum levels with TRM, 25-hydroxyvitamin-D3 and 1,25-dihydroxyvitamin-D3 serum levels for each of the six time intervals were compared between patients who experienced TRM and those that did not die or died from relapse (*n* = 143, 23 with TRM, Table 2, Table S1). For this, we used the nonparametric Mann–Whitney U test and a significance level Bonferroni-corrected for the multiple testing (0.05/(6 × 2) = 0.004, two metabolites, six time intervals). Lower late follow-up 25-hydroxyvitamin-D3 and lower peritransplant 1,25-dihydroxyvitamin-D3 levels were statistically significantly associated with increased TRM (Fig. 1c, d; *P* = 0.002 or *P* = 0.001, respectively). For all other time intervals, there was no statistically significant difference of serum levels between the two groups (*P* > 0.004). We also used nonparametric Mann–Whitney U to test for an association of 1,25(OH)2D3 serum levels and occurrence of severe aGvHD or chronic GvHD (cGvHD). However, no statistically significant association between the groups was detected (Supplemental Fig. 1).

### Discovery cohort substantiated peritransplant 1,25-dihydroxyvitamin-D3 levels to predict first-year TRM independent of severe aGvHD

In order to understand the robustness of discovery cohort findings upon adjustment for other risk factors and to incorporate time-to-death, we evaluated the detected significant associations further by a Cox-regression analysis and found the following (Table 3): (i) for late follow-up 25-hydoxyvitamin-D3, the significant association with TRM in the unadjusted model (*n* = 88, 17 with TRM, *P* = 0.001, hazard ratio (HR) = 0.98 per unit increase) persisted when adjusting for age, sex, donor status, and tumor stage (*n* = 77, 16 with TRM, *P* = 0.004, HR = 0.98), but diminished when adding severe aGvHD into the model (*n* = 77, 16 with TRM, *P* = 0.069, HR = 0.99). (ii) For peritransplant 1,25-dihydroxyvitamin-D3, the significant association with TRM observed in the unadjusted model (*n* = 143, 23 with TRM, *P* = 0.001, HR = 0.99) persisted also after accounting for severe aGvHD (*n* = 128, 21 with TRM, *P* = 0.005, HR = 0.99). This association of 1,25-dihydroxyvitamin-D3 with TRM was also independent of peritransplant 25-hydroxyvitamin-D3 levels (*n* = 128, 21 with TRM, *P* = 0.005, HR = 0.99). In summary, we selected the peritransplant 1,25-dihydroxyvitamin-D3 levels as the predominantly interesting, since this association was independent of severe aGvHD and measured around the time of transplantation, while the late 25-hydroxyvitamin-D3 levels appeared to be observed together with the occurrence of severe aGvHD (median time to maximum severe aGvHD across all patients day 70, interquartile range 53–135 days, Table 2).

**Table 3:** Association of late follow-up 25-hydroxyvitamin-D3 and peritransplant 1,25-dihydroxyvitamin-D3 levels with TRM.

Model	25(OH)D3 late follow-up (day 32 to 100)			1,25(OH)2D3 peritransplant (day −2 to 7)		
	#at risk/ #TRM	HR (95% CI)	*P*	#at risk/#TRM	HR (95% CI)	*P*
Unadjusted	88/17			143/23		
Serum level		0.98 (0.97; 0.99)	**0.001**		0.99 (0.99; 1.00)	**0.001**
Adjusted I	77/16			128/21		
Serum level	[nM]	0.98 (0.97; 0.99)	**0.004**	[pM]	0.99 (0.98, 1.00)	**0.002**
Age [yr]		1.08 (1.00; 1.16)	**0.030**		1.08 (1.02; 1.15)	**0.013**
Male sex		0.57 (0.19; 1.71)	0.314		0.45 (0.18; 1.13)	0.089
Unrelated donor^a^		0.61 (0.20; 1.89)	0.390		1.04 (0.39; 2.82)	0.935
Late tumor stage^b^		1.81 (0.66; 4.98)	0.250		1.82 (0.75; 4.41)	0.186
Adjusted II	77/16			128/21		
Serum level	[nM]	0.99 (0.98, 1.00)	0.069	[pM]	0.99 (0.96; 1.00)	**0.005**
Age [yr]		1.05 (0.98; 1.13)	0.139		1.05 (0.99; 1.12)	0.087
Sex		0.86 (0.26; 2.81)	0.803		1.40 (0.48; 4.07)	0.538
Unrelated donor^a^		0.53 (0.16; 1.72)	0.287		0.86 (0.30; 2.45)	0.774
Late tumor stage^b^		2.04 (0.72; 5.84)	0.182		1.72 (0.68; 4.31)	0.250
Severe aGvHD^c^		4.56 (1.35; 15.39)	**0.015**		14.81 (5.09; 43.08)	**<0.0005**
Adjusted III	77/16			128/21		
Serum level	[nM]	0.99 (0.96, 1.00)	0.068	[pM]	0.99 (0.99; 1.00)	**0.005**
Age [yr]		1.05 (0.98; 1.13)	0.144		1.05 (0.99; 1.12)	0.090
Sex		0.86 (0.26; 2.82)	0.808		1.37 (0.46; 4.12)	0.576
Unrelated donor^a^		0.52 (0.16; 1.72)	0.286		0.85 (0.29; 2.44)	0.756
Late tumor stage^b^		2.03 (0.70; 5.88)	0.194		1.73 (0.69; 4.34)	0.247
Severe aGvHD^c^		4.56 (1.35; 15.44)	**0.015**		14.81 (5.08; 43.13)	**<0.0005**
25(OH)D3 d-2–7^d^	[nM]	1.00 (0.97; 1.03)	0.929	[pM]	1.00 (0.98; 1.02)	0.871

Shown are results from Cox proportional hazards models for the association of late follow-up 25-hydroxyvitamin-D3 (25(OH)D3, day 32 to 100) or peritransplant 1,25-dihydroxyvitamin-D3 (1,25(OH)2D3, day −2 to 7) with TRM without and with adjustment for risk factors and sensitivity analyses. *P* values ≤0.05 are marked in bold.

*TRM* treatment-related mortality, *25(OH)D3* 25-hydroxyvitamin-D3, *1,25(OH)2D3* 1,25-dihydroxyvitamin-D3, *HR* hazard ratio, *aGvHD* acute Graft-versus-Host disease.

^a^Patients with unrelated donors versus patients with sibling donors.

^b^Classification according to EBMT risk score [50] late stage versus early/intermediate stage.

^c^Any occurrence of aGvHD 3–4 [14] within 1 year after HSCT versus no occurrence of aGvHD 3–4.

^d^Peritransplant (day −2 to 7) 25-hydroxyvitamin-D3.

### Clinical relevance and optimal threshold for 1,25-dihydroxyvitamin-D3 to predict 1-year TRM

**Fig. 2:**
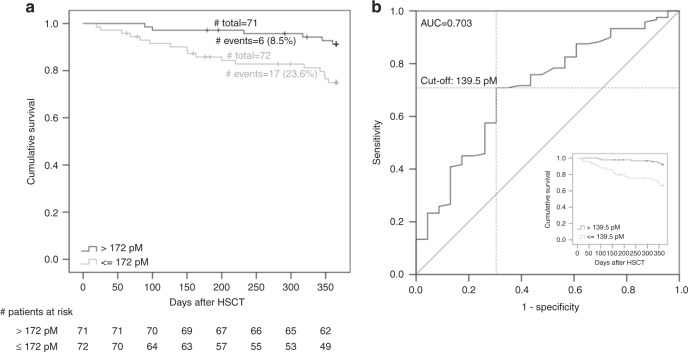
Treatment-related survival and receiver-operating-characteristics curve comparing patients with high versus low peritransplant 1,25-dihydroxyvitamin-D3 serum levels in the discovery cohort. **a** Shown is the Kaplan–Meier curve comparing patients above versus below median peritransplant 1,25-dihydroxyvitamin-D3 serum levels (day −2 to 7, median = 172 pM). **b** Also shown is the Receiver-Operating-Characteristics (ROC) curve for the ability of peritransplant 1,25-dihydroxyvitamin-D3 serum levels to predict TRM. The best cutoff calculated by Youden-Index [15] was derived as 139.5 pM. Embedded in **b** is the Kaplan–Meier curve using this best cutoff rather than the median in the discovery cohort.

We visualized the differential survival between patients with high versus low peritransplant 1,25-dihydroxyvitamin-D3 levels (dichotomized at the median 172 pM, Fig. 2a) by Kaplan–Meier curves. We found a statistical significant association of the dichotomized 1,25-dihydroxyvitamin-D3 levels with time-to-death (median time-to-death=151 days vs. 275 days in the group ≤172 pM vs. >172 pM, respectively; log rank test *P* = 0.009, unadjusted Cox-model *P* = 0.013, HR = 3.24; Cox-model adjusted for risk factors including severe aGvHD *P* = 0.006, HR = 4.81), which was consistent with the finding for quantitatively modeled 1,25-dihydroxyvitamin-D3 association with the binary outcome TRM reported above. We found the cutoff at 139.5 pM to be the best to separate those at high risk of TRM from those at low risk yielding an AUC of 0.703 (Fig. 2b). All further analyses were conducted with 1,25-dihydroxyvitamin-D3 levels dichotomized at this optimal cutoff.

### Replication cohorts support the predictive value of peritransplant 1,25-dihydroxyvitamin-D3 for 1-year TRM independent of severe aGvHD

**Fig. 3:**
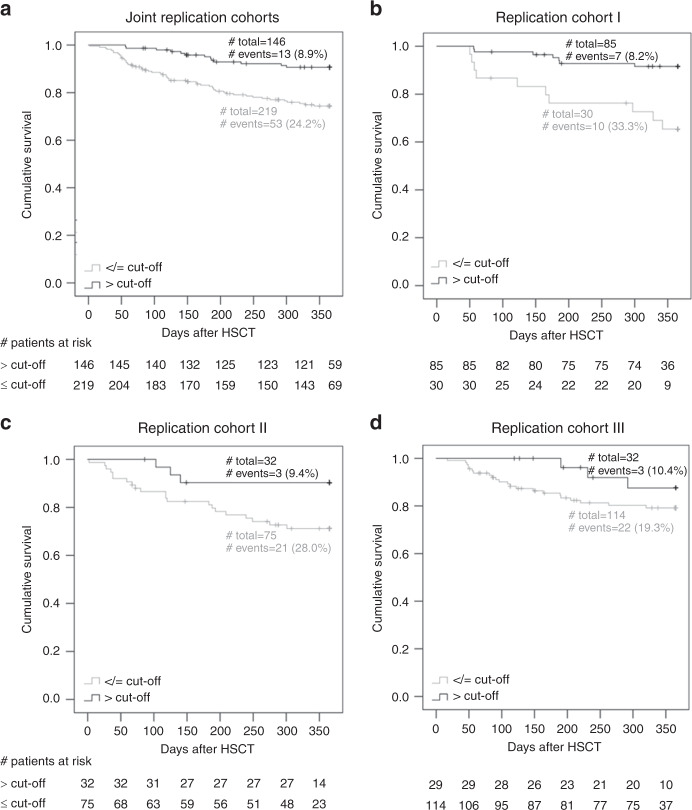
Treatment-related survival in the replication cohort comparing patients with peritransplant 1,25-dihydroxyvitamin-D3 serum levels above and below the cutoff 139.5 pM. Shown are Kaplan–Meier curves comparing patients with peritransplant 1,25-dihydroxyvitamin-D3 serum levels (day −2 to 7) above versus below the cutoff 139.5 pM in **a** the joint replication cohorts, **b** replication cohort I, **c** replication cohort II, and **d** replication cohort III.

To replicate our finding from the discovery cohort, we collected serum for the relevant time interval, peritransplant, from three independent replication cohorts. Peritransplant 1,25-dihydroxyvitamin-D3 and, for comparison, also peritransplant 25-hydroxyvitamin-D3 were measured yielding an analyzable replication dataset of 365 patients. Our replication stage was designed to replicate our initial finding and to generalize to other clinical settings. Of note, the type of vitamin D3 supplementation varied between the three cohorts: same high-dose supplementation as in the discovery cohort (replication cohort I), moderate supplementation (replication cohort II), or no supplementation (replication cohort III). The level of supplementation was reflected in the cohort-specific levels of peritransplant 1,25-dihydroxyvitamin-D3 and 25-hydroxyvitamin-D3 (Table 2). When separating the 365 patients in the joint replication data by the optimal cutoff for 1,25-dihydroxyvitamin-D3 at 139.5 pM, we found a larger proportion of TRM among patients with low vs. high levels (24.2% vs. 8.9%, Fig. 3a). This was also observed for each of the three replication cohorts separately (33.3% vs. 8.2%, 28.0% vs. 9.4%, 19.4% vs. 10.3%, respectively, Fig. 3b-d). We also found clearly separated Kaplan–Meier survival curves in each of the replication cohorts and overall.

When applying Cox proportional hazards models to the joint replication data (Table 4), we found statistically significant association for low 1,25-dihydroxyvitamin-D3 with increased one-year TRM that was very robust upon different approaches for adjustment (without any covariates, adjusting for risk factors excluding severe aGvHD or for risk factors including severe aGvHD: *P* < 0.0005, HR = 3.51; *P* < 0.0005, HR = 3.30; *P* = 0.001, HR = 3.31, respectively). Of note, adjusting for the Karnofsky performance score (KPS) at time of transplantation did not change the statistically significant association between 1,25-dihydroxyvitamin-D3 and one-year TRM (results not shown).

**Table 4:** Association of peritransplant 1,25-dihydroxyvitamin-D3 levels dichotomized at 139.5 pM with TRM in discovery cohort and in joint replication cohorts.

	Peritransplant 1,25(OH)2D3 discovery data			Peritransplant 1,25(OH)2D3 joint replication data		
	#at risk/#TRM	HR (95% CI)	*P*	#at risk/#TRM	HR (95% CI)	*P*
Unadjusted	143/23			365/66		
Serum level ≤139.5 pM^a^		5.16 (2.12; 12.57)	<**0.0005**		3.51 (1.79; 6.88)	<**0.0005**
Adjusted I	128/21			364/66		
Serum level ≤139.5 pM^a^		6.10 (2.24; 16.63)	**<0.0005**		3.30 (1.70; 6.44)	**<0.0005**
Age [yr]		1.08 (1.01; 1.15)	**0.018**		1.05 (1.02; 1.07)	**0.001**
Male sex		0.45 (0.19; 1.10)	0.081		0.92 (0.55; 1.54)	0.749
Unrelated donor^b^		0.99 (0.39; 2.54)	0.986		2.77 (1.25; 6.13)	**0.012**
Late tumor stage^c^		1.81 (0.073; 4.46)	0.197		1.03 (0.61; 1.72)	0.919
Adjusted II	128/21			361/63		
Serum level ≤139.5 pM^a^		6.00 (2.16; 16.63)	**0.001**		3.31 (1.68; 6.53)	**0.001**
Age [yr]		1.05 (0.99; 1.12)	0.134		1.04 (1.01; 1.07)	**0.006**
Male sex		1.21 (0.44; 3.33)	0.712		0.95 (0.56; 1.61)	0.848
Unrelated donor^b^		0.82 (0.31; 2.21)	0.700		2.49 (1.11; 5.56)	**0.026**
Late tumor stage^c^		1.63 (0.63; 4.26)	0.317		0.99 (0.58; 1.69)	0.960
Severe aGvHD^d^		16.18 (5.56; 47.07)	**<0.0005**		4.96 (2.91; 8.47)	<**0.0005**

Shown are results from Cox proportional hazards models based on the 143 patients (23 TRM) of the discovery cohort and 365 patients (66 TRM) combining the three replication cohorts. Models for the joint replication cohorts are stratified by cohort. *P* values ≤0.05 are marked in bold.

*TRM* treatment-related mortality, *1,25(OH)2D3* 1,25-dihydroxyvitamin-D3, *HR* hazard ratio, *aGvHD* acute Graft-versus-Host disease.

^a^1,25(OH)2D3 level d-2–7 ≤/> cutoff 139.5 pM.

^b^Patients with unrelated donors versus patients with sibling donors.

^c^Classification according to EBMT risk score [50] late stage versus early/intermediate stage.

^d^Any occurrence of aGvHD 3–4 [14] within 1 year after HSCT versus no occurrence of aGvHD 3–4.

To address the question whether absolute levels of peritransplant 1,25-dihydroxyvitamin-D3 are responsible for the outcome or whether peritransplant 1,25-dihydroxyvitamin-D3 levels are a surrogate marker for another biological process, we normalized peritransplant 1,25-dihydroxyvitamin-D3 levels to peritransplant 25-hydroxyvitamin-D3 levels. The discrimination between patients with and without TRM disappeared in the replication datasets I–III (Supplemental Fig. 2). This indicates that the absolute levels of 1,25-dihydroxyvitmain-D3 are indeed crucial. However, in the discovery cohort the discrimination between patients with and without TRM was still possible after normalization. Based on these data it is not completely clear whether the absolute levels alone are responsible for the outcome.

### Subgroup analysis for patients with severe aGvHD and those without severe aGvHD

**Fig. 4:**
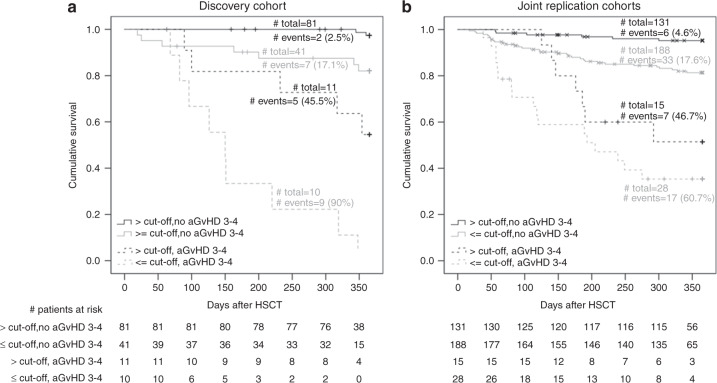
Treatment-related survival separated by severe aGvHD status in discovery cohort and joint replication cohorts comparing patients with peritransplant 1,25-dihydroxyvitamin-D3 serum levels above and below the cutoff 139.5 pM. Shown are Kaplan–Meier curves comparing patients with peritransplant 1,25-dihydroxyvitamin-D3 serum levels (day −2 to 7) above versus below the cutoff 139.5 pM separately for the group of patients without severe aGvHD (aGvHD 3–4) and the group of patients with severe aGvHD **a** in the discovery cohort and **b** in the joint replication cohorts.

We followed the finding that low 1,25-dihydroxyvitamin-D3 predicted TRM independent of severe aGvHD by an analysis separated by aGvHD status. We found shorter median time-to-death and higher Cox-model-derived HR estimates for patients with low vs. high 1,25-dihydroxyvitamin-D3 levels in all four groups (Table S2): (1) in patients without severe aGvHD in the discovery (163 days vs. 353 days comparing patients ≤139.5 pM vs. patients >139.5 pM; HR = 7.83, *P* = 0.010), (2) in patients without severe aGvHD in the joint replication data (122 days vs. 144 days; HR = 5.28, *P* = 0.001), (3) in patients with severe aGvHD in the discovery (150 days vs. 232 days; HR = 5.43, *P* = 0.006), and (4) in patients with severe aGvHD in the joint replication data (113 days vs. 176 days; HR = 2.06, *P* = 0.135). Given the consistent directionality in the difference in median time-to-death and in HR estimates across these four groups, the lack of statistical significance in some of the groups may be due to the low number of patients per group and thus limited power to detect effects group-specifically. The consistent pattern across the four groups can be visualized in Kaplan–Meier-curves (Fig. 4). This supports the notion that the higher mortality for patients with low vs. high 1,25-dihydroxyvitamin-D3 levels is apparent in patients with severe aGvHD as well as those without, which is in line with the previous results that the effect was independent from severe aGvHD as shown in the models adjusting for severe aGvHD.

## Discussion

Our data of altogether 508 patients undergoing HSCT highlighted higher 1,25-dihydroxyvitamin-D3 levels at the time of HSCT to be a strong predictor for one-year TRM independent of severe aGvHD. This predictor was independent of baseline characteristics known to affect TRM and independent of 25-hydroxyvitamin-D3 levels. We identified the optimal threshold of 139.5 pM 1,25-dihydroxyvitamin-D3 to detect patients at high risk for TRM and replicated our finding in independent replication data consisting of 365 patients from three different clinical settings.

To our knowledge, our present study is the first study on 1,25-dihydroxyvitamin-D3 in the context of allogeneic HSCT and with 508 patients the largest study on vitamin D metabolites in the context of allogeneic HSCT. Up to now, all published studies on vitamin D included less patients [17, 18].

Existing studies on vitamin D in the context of allogeneic HSCT are dealing with the role of 25-hydroxyvitamin-D3, the precursor of 1,25-dihydroxyvitamin-D3. A number of reports has described insufficiency for 25-hydroxyvitamin-D3 in patients undergoing allogeneic HSCT [2, 5, 6, 19]. As a consequence, we and others used different approaches to increase vitamin D levels in patients undergoing HSCT [7, 12, 20–22]. At present, the role of 25-hydroxyvitamin-D3 for survival after HSCT has been examined in eight studies [3–5, 7, 18, 23–25]. In four studies early low 25-hydroxyvitamin-D3 levels were associated with increased overall mortality [4, 5, 18, 25]. In contrast, no significant impact of early 25-hydroxyvitamin-D3 serum levels on overall survival was described in four studies [3, 7, 23, 24]. In line, also in our study low peritransplant 25-hydroxyvitamin-D3 levels were no risk factor for mortality. However, the primary endpoint of our analysis was TRM, not overall mortality. Conflicting data have also been published looking at 25-hydroxyvitamin-D3 and association with aGvHD [5, 23, 26] and cGvHD [4, 5, 7, 26–28]. When we tested for an association of severe aGvHD or cGvHD with 1,25(OH)2D3 levels, respectively, no significant association was detected.

We found that the association of low late follow-up 25-hydroxyvitamin-D3 levels with TRM vanished upon accounting for severe aGvHD in the Cox-model. Since severe aGvHD is a major risk factor for TRM [29], one might speculate, that the decreased 25-hydroxyvitamin-D3 levels we observed in the late follow-up of patients with TRM were—at least in part—due to severe aGvHD, which is characterized by gastrointestinal tract involvement and often includes diarrhea [29]. Indeed, also Wallace et al. found the association of 25-hydroxyvitamin-D3 deficiency 100 days post-HSCT with reduced mortality in pediatric patients to be a consequence of aGvHD rather than vitamin D deficiency per se [5].

In contrast to 25-hydroxyvitamin-D3, the association of peritransplant 1,25-dihydroxyvitamin-D3 levels with TRM both in the discovery and in the replication data was also robust upon accounting for factors known to be critical for TRM, including severe aGvHD. No study up to now has addressed the role of 1,25-dihydroxyvitamin-D3 in HSCT. In renal transplantation (Tx) low 1,25-dihydroxyvitamin-D3 levels have been reported to be associated with increased mortality [30]. Others observed low 1,25-dihydroxyvitamin-D3 levels to be indicative for graft rejection and/or infections after renal Tx [31, 32]. However, since 1,25-dihydroxyvitamin-D3 is produced in the kidney, low levels of 1,25-dihydroxyvitamin-D3 during renal Tx might be indicative for reduced renal function [30]. Low postoperative (until d21 after Tx) 1,25-dihydroxyvitamin-D3 concentrations were also shown to be independent risk factors for 1-year mortality following cardiac Tx [33].

Whether these observations implicate a causal relationship between low 1,25-dihydroxyvitamin-D3 and adverse Tx outcome or whether the high 1,25-dihydroxyvitamin-D3 is indicative for a better general constitution of a patient remains unclear. There is some evidence of a causal relationship: the administration of 1,25-dihydroxyvitamin-D3 in rat liver Tx models reduced mortality and graft rejection [34, 35]. Similarly, Pakkala et al. observed an analog of 1,25-dihydroxyvitamin-D3 to prevent aGvHD in a rat model for HSCT [10]. Further evidence for a causal link is provided by the fact that vitamin D receptor (VDR) gene polymorphisms are associated with development of aGvHD in humans [11]. In line, a recent study reported a decreased incidence of cGvHD upon vitamin D supplementation of patients with a certain VDR genotype [12]. Interestingly, VDR gene polymorphisms have also been characterized as risk factors for graft rejection after liver Tx [36]. Zhang et al. showed that the beneficial effect of 1,25-dihydroxyvitamin-D3 in rat liver Tx involves the reduction of proinflammatory pathways [34] pointing to an active immunomodulatory role of 1,25-dihydroxyvitamin-D3 in improving the outcome. Consistently, administration of 1,25-dihydroxyvitamin-D3 or an analogon to patients receiving renal Tx was found to reduce the risk of adverse events [31, 37].

A major question arising from our study is, why patients with low peritransplant 1,25-dihydroxyvitamin-D3 levels are at 3.3-fold risk for TRM within 1 year compared to patients with high levels in our data. One possibility might be that a high 1,25-dihydroxyvitamin-D3 level represents a marker for a patient’s better overall condition. On the other hand, high 1,25-dihydroxyvitamin-D3 levels might actively mediate a beneficial functional effect. We tried to address this question by normalizing the peritransplant 1,25-dihydroxyvitamin-D3 levels to peritransplant 25-hydroxyvitamin-D3 levels but did not receive a clear result. In this context it needs to be emphasized that the differences in 1,25-dihydroxyvitamin-D3 levels observed in our study are not a result of impaired renal function in some patients since renal function of our patients was tightly controlled. Furthermore, our finding was robust upon adjusting for baseline factors displaying the overall condition of patients like age, conditioning or tumor stage prior to HSCT. Importantly, 1,25-dihydroxyvitamin-D3 is not limited to renal production: also immune cells, especially myeloid cells are known to produce the active vitamin D metabolite and thereby contribute to local immune regulation [9, 38]. The early peak in 1,25-dihydroxyvitamin-D3 levels could—at least in part—be due to increased macrophage activity and could be indicative for patients harboring macrophage populations with primarily immune regulatory capacities. Another possibility could be that high circulating concentrations of 1,25-dihydroxyvitamin-D3 at the time of HSCT directly modulate one or several critical factors known to be crucial for HSCT outcome: 1,25-dihydroxyvitamin-D3 has been demonstrated to drive differentiation of monocytes during hematopoiesis [39]. Interestingly, higher levels of monocytes following HSCT have been correlated with better overall survival [40–42]. In light of these findings one could speculate that the availability of 1,25-dihydroxyvitamin-D3 at the time of HSCT improves the development of monocytes from the graft, which is beneficial for patient survival. Furthermore, also remaining immune cells in the recipient might be affected by high 1,25-dihydroxyvitamin-D3 levels around HSCT since 1,25-dihydroxyvitamin-D3 has been shown e.g., to tolerize dendritic cells and to induce regulatory T cells [9, 43–45] leading to a more immunosuppressive environment in the patient. A critical risk factor for TRM is severe aGvHD, which predominantly affects the gut. Although aGvHD typically develops during the follow-up, early events around the time of HSCT, as conditioning-induced damage of the intestinal barrier or microbial changes due to conditioning or use of prophylactic antibiotics, are known to be critical for the development of GvHD [46, 47]. A role of 1,25-dihydroxyvitamin-D3 for gut homeostasis is supported by murine models, showing that VDR -/- mice exhibit significant shifts in the microbiota relative to control mice [48] and VDR signaling preserves the intestinal epithelial barrier integrity [49]. Therefore, high 1,25-dihydroxyvitamin-D3 levels around HSCT may strengthen the intestinal epithelial barrier function in HSCT patients and exert immunoregulatory effects [44, 45] thereby possibly ameliorating gut GvHD. In case of a direct effect of high 1,25-dihydroxyvitamin-D3 levels at the time of HSCT, pretransplant supplementation of HSCT patients with vitamin D metabolites would be highly recommended as supportive therapy.

A limitation of the current study is that retrospective studies are unable to correct for changes in practice at each facility over time. Each cohort was transplanted at different times and under different protocols. Furthermore, historical controls are used and there are limitations to using such controls for studies. However, a clear strength of our data are our independent replication stage that replicates the initial finding and generalizes the effect to other clinical settings including other modes of vitamin D supplementation or other clinical approaches towards concomitant therapy. This overcomes the potentially perceived limitation of the discovery cohort, that the high-dose vitamin D3 supplementation administered to the discovery cohort patients is not standard care of HSCT patients. While each of the replication cohorts is not large and thus we refrained from statistical testing by replication cohort separately, the joint replication data with 365 HSCT patients are substantial and results are stable between discovery and replication as well as across different analyses approaches and models with different baseline risk factor control. Therefore, our data suggests 1,25-dihydroxyvitamin-D3 at the time of HSCT as a predictive factor for TRM.

In summary, our data highlight the apparently underestimated role of 1,25-dihydroxyvitamin-D3 and its value to predict outcome of allogeneic HSCT. Whether supplementation with 1,25-dihydroxyvitamin-D3 or 1,25-dihydroxyvitamin-D3 analogs could improve patient survival needs to be addressed in future prospective studies.

## Supplementary information

Supplemental Material
