# Supplementary material for: 1,25-dihydroxyvitamin-D3 but not the clinically applied marker 25-hydroxyvitamin-D3 predicts survival after stem cell transplantation
Source: Bone Marrow Transplant. 2020 Aug 27;56(2):419–33. doi: 10.1038/s41409-020-01031-w (PMC7870805; doi:10.1038/s41409-020-01031-w)
Supplement: Supplementary file 1 — Supplemental Material [file 41409_2020_1031_MOESM1_ESM.pdf]

Supplemental Material Peter et al.

Supplemental Table S1: Sample size, 25-hydroxyvitamin-D3 and 1,25-dihydroxyvitamin-D3 serum levels and their association with TRM for each time interval in the discovery cohort. P-values are from Mann Whitney U test testing for difference in 25-hydroxyvitamin-D3 (25(OH)D3) and 1,25-dihydroxyvitamin-D3 (1,25(OH)2D3) levels between patients who died due to TRM vs. those who did not (i.e. did not die or died from other cause). P-values ≤0.05 (nominal significance) or ≤0.05/(6x2)=0.004 (Bonferroni-corrected significance level) are marked in bold.

**mortality/ serum values/ association**	**days**						
	**-16 to -6**	**-2 to 7**	**11 to 17**	**18 to 24**	**25 to 31**	**32 to 100**	**101 to 365**
# at risk*	143	143	143	143	142	141	133
mortality†							
TRM	0	0	0	1	1	6	15
death, other cause	0	0	0	0	0	2	0
lost-to-follow-up	0	0	0	0	0	0	0
# severe aGvHD‡	0	0	1	1	1	11	7
**25(OH)D3 values**							
n with measurement							
overall	143	143	120	106	41	88	-
with TRM	23	23	17	16	5	17	
without TRM	120	120	103	90	36	61	
median [nM]							
overall	43.5	54.0	77.0	91.0	109.0	146.0	-
with TRM	43.5	49.0	67.0	90.0	124.0	74.5	
without TRM	44.5	54.5	77.0	91.0	107.1	150.5	
P-Value	0.620	0.865	0.901	0.701	0.802	**0.002**	-
**1,25(OH)2D3 values**							
n with measurement							
overall	143	143	120	106	41	88	-
with TRM	23	23	17	16	5	17	
without TRM	120	120	103	90	36	71	
median [pM]							
overall	82.0	172.0	122.0	94.0	79.0	89.0	-
with TRM	64.0	106.0	71.0	104.4	111.0	98.0	
without TRM	82.0	180.9	124.0	90.0	77.5	88.0	
P-Value	**0.033**	**0.001**	0.068	0.338	0.360	0.475	-

TRM=treatment-related mortality, aGvHD=acute Graft-versus-Host disease

*at start of time interval; †during time interval; ‡time of recorded maximum aGvHD 3-4 (Marks et al., Blood, 2008) within this interval

Supplemental Figure 1: Time trend of 1,25-dihydroxyvitamin-D3 serum levels and association with severe acute GvHD (aGvHD) and chronic GvHD (cGvHD) in the discovery cohort. Shown are 1,25-dihydroxyvitamin-D3 (1,25(OH)2D3) serum levels and their association to severe aGvHD **(A)** and cGvHD **(B)** in the discovery cohort. Serum levels were measured repeatedly at hospital admission (baseline, day -16 to -6), peritransplant (day -2 to 7), during the weekly early follow-up (day 11 to 17, 18 to 24, 25 to 31) and the late follow-up (day 32 to 100 after HSCT). Also shown is the number of available patients with measurement of 1,25(OH)2D3 levels per time interval separately for patients with or without (A) severe aGvHD (n=122 with aGvHD 0/1/2; n=21 with aGvHD 3/4) **(B)** cGvHD (n=72 with cGvHD; n=44 without cGvHD; missing information on cGvHD status for 27 patients). Median serum levels are stated in bold for each boxplot; testing for difference of serum levels between the two groups was performed using Mann Whitney U Test. No significant differences in 1,25(OH)2D3 serum levels between patients with and without aGvHD or cGvHD were detected.

A

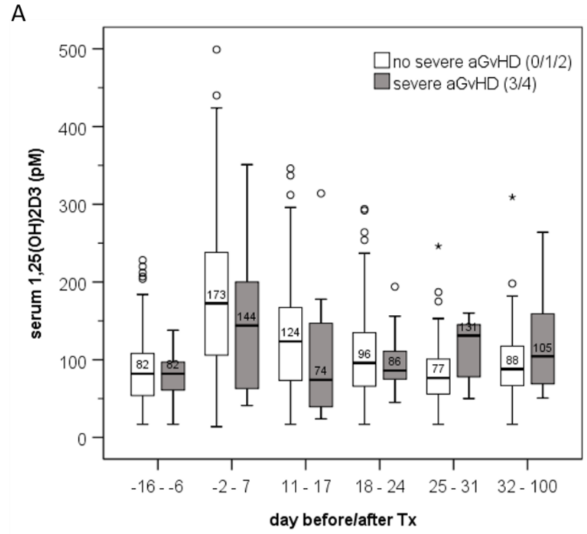

	days					
	-16 to -6	-2 to 7	11 to 17	18 to 24	25 to 31	32 to 100
**n with 1,25(OH)2D3 measurement**						
overall	143	143	120	106	41	88
aGvHD 0/1/2	122	122	104	89	34	72
aGvHD 3/4	21	21	16	17	7	16

B

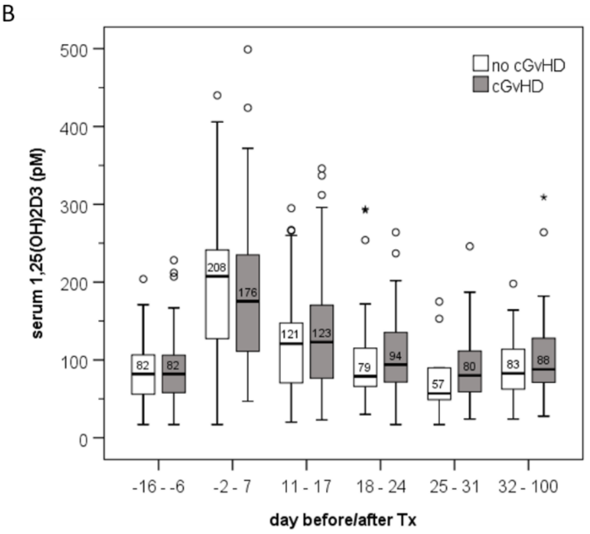

	days					
	-16 to -6	-2 to 7	11 to 17	18 to 24	25 to 31	32 to 100
**n with 1,25(OH)2D3 measurement**						
overall	116	116	99	90	33	69
no cGvHD	44	44	40	34	9	28
cGvHD	72	72	59	56	24	41

Supplemental Figure 2: Normalization of peritransplant 1,25-dihydroxyvitamin-D3 serum levels to peritransplant 25-hydroxvitamin-D3 levels and association with transplant-related mortality in the different cohorts. Shown are normalized 1,25-dihydroxyvitamin-D3 (1,25(OH)2D3) serum levels and their association with one-year TRM in the different cohorts (discovery data: no TRM n=120; TRM n=23; replication data I: no TRM n=40; TRM n=8; replication data II: no RM n=57; TRM n=12; replication data III: no TRM n=116; TR n=25). Median normalized levels are stated in bold for each boxplot; testing for difference of serum levels between the two groups was performed using Mann Whitney U Test.

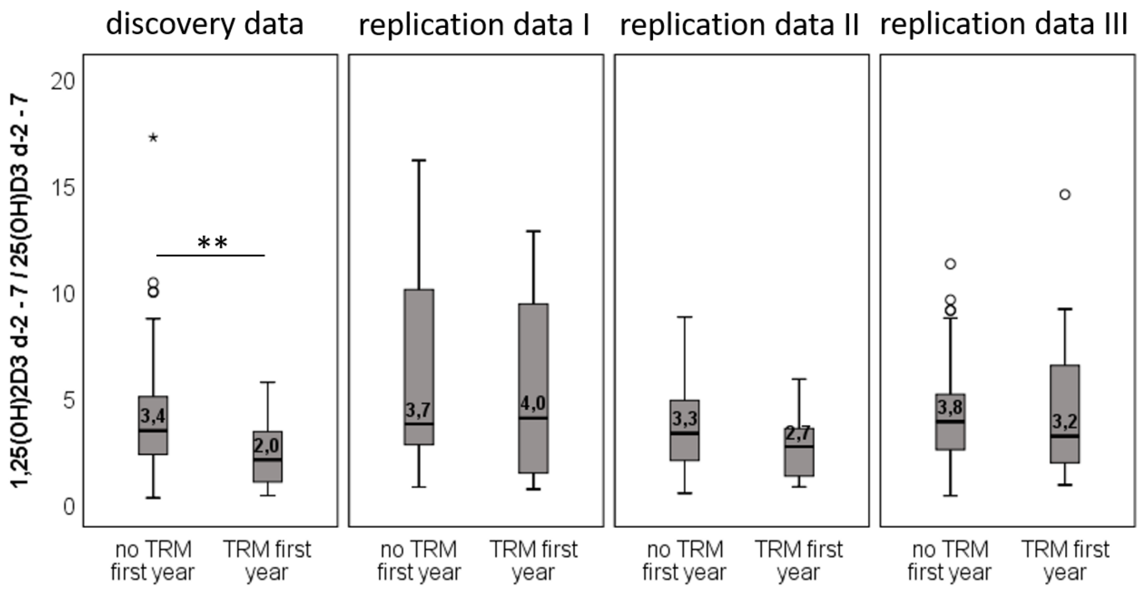

Supplemental Table S2: **Details on the association of peritransplant 1,25-dihydroxyvitamin-D3 levels with TRM in the replication cohorts dichotomized at the cut-off 139.5 pM and separated into patients with and without severe aGvHD**. Shown are results from Cox proportional hazards models based on the 143 patients (23 TRM) of the discovery data and 362 patients (aGvHD not evaluable in 3 patients, 63 TRM) combining the three replication data sets. Models for the joint replication data are stratified by cohort. P values ≤0.05 are marked in bold.

	discovery cohort			joint replication cohorts		
	**#at risk/ #TRM**	**HR (95% CI)**	**P**	**#at risk/ #TRM**	**HR (95% CI)**	**P**
**without severe aGvHD***						
**unadjusted**	122/9			319/39		
serum level ≤139.5 pM†		7.83 (1.62; 37.70)	**0.010**		5.28 (2.02; 13.77)	**0.001**
**adjusted I**	108/8			318/39		
serum level ≤139.5 pM‡		6.32 (1.09; 36.72)	**0.040**		4.67 (1.81; 12.05)	**0.001**
age [yr]		1.10 (0.99; 1.23)	0.080		1.05 (1.01; 1.08)	**0.008**
male sex		0.85 (0.15; 4.74)	0.857		0.83 (0.42; 1.63)	0.588
unrelated donor‡		1.10 (0.24; 5.05)	0.906		2.34 (0.90; 6.09)	0.081
late tumor stage§		1.76 (0.41; 7.61)	0.450		0.81 (0.40; 1.64)	0.549
**with severe aGvHD***						
**unadjusted** serum level ≤139.5 pM†	20/14	5.43 (1.61; 18.31)	**0.006**	43/24	2.06 (0.80; 5.33)	0.135
**adjusted I**	19/13			43/24		
serum level ≤139.5 pM‡		6.23 (1.51; 25.67)	0.011		2.11 (0.70; 6.38)	0.187
age [yr]		1.00 (0.92; 1.10)	0.980		1.03 (0.98; 1.08)	0.260
male sex		1.85 (0.46; 7.40)	0.385		1.20 (0.50;2.90)	0.686
unrelated donor‡		1.00 (0.22; 4.42)	0.995		2.44 (0.47; 12.58)	0.288
late tumor stage§		1.99 (0.56; 7.12)	0.290		1.07 (0.41; 2.79)	0.886

TRM=treatment-related mortality, aGvHD=acute Graft-versus-Host disease; HR=hazard ratio

*any occurrence of aGvHD 3-4 (Marks et al., Blood, 2008) within one year after HSCT versus no occurrence of aGvHD 3-4; one patient in discovery cohort was excluded from analysis due to short follow-up time period

†1,25(OH)2D3 level d-2 – 7 ≤/> cut-off 139.5 pM

‡patients with unrelated donors versus patients with sibling donors

§classification according to EBMT risk score (Gratwohl A., BMT, 2012) late stage versus early/intermediate stage
